# Evaluation of prestyloid recess morphology and ulnar-sided contrast leakage in CT arthrography of the wrist

**DOI:** 10.1186/s12891-022-05241-9

**Published:** 2022-03-24

**Authors:** Carsten Herbert Gietzen, Andreas Steven Kunz, Karsten Sebastian Luetkens, Henner Huflage, Georgios Christopoulos, Jörg van Schoonhoven, Thorsten Alexander Bley, Rainer Schmitt, Jan-Peter Grunz

**Affiliations:** 1 Department of Diagnostic and Interventional Radiology, University Hospital Würzburg, Oberdürrbacher Straße 6, 97080 Würzburg, Germany; 2 Department of Diagnostic and Interventional Radiology, Rhön-Klinikum Campus Bad Neustadt, Von-Guttenberg-Straße 11, 97616 Bad Neustadt an der Saale, Germany; 3 Department of Hand Surgery, Rhön-Klinikum Campus Bad Neustadt, Von-Guttenberg-Straße 11, 97616 Bad Neustadt an der Saale, Germany; 4 Department of Radiology, University Hospital LMU Munich, Marchioninistraße 15, 81377 Munich, Germany

**Keywords:** Prestyloid recess, Triangular fibrocartilage complex, Tomography, X-ray computed, Arthrography

## Abstract

**Background:**

In wrist arthrograms, aberrant contrast material is frequently seen extending into the soft tissue adjacent to the ulnar styloid process. Since the prestyloid recess can mimic contrast leakage in CT arthrography, this study aims to provide a detailed analysis of its morphologic variability, while investigating whether actual ulnar-sided leakage is associated with injuries of the triangular fibrocartilage complex (TFCC).

**Methods:**

Eighty-six patients with positive wrist trauma history underwent multi-compartment CT arthrography (40 women, median age 44.5 years). Studies were reviewed by two board-certified radiologists, who documented the morphology of the prestyloid recess regarding size, opening type, shape and position, as well as the presence or absence of ulnar-sided contrast leakage. Correlations between leakage and the presence of TFCC injuries were assessed using the mean square contingency coefficient (r_ɸ_).

**Results:**

The most common configuration of the prestyloid recess included a narrow opening (73.26%; width 2.26 ± 1.43 mm), saccular shape (66.28%), and palmar position compared to the styloid process (55.81%). Its mean length and anterior–posterior diameter were 6.89 ± 2.36 and 5.05 ± 1.97 mm, respectively. Ulnar-sided contrast leakage was reported in 29 patients (33.72%) with a mean extent of 12.30 ± 5.31 mm. Leakage occurred more often in patients with ulnar-sided TFCC injuries (r_ɸ_ = 0.480; *p* < 0.001), whereas no association was found for lesions of the central articular disc (r_ɸ_ = 0.172; *p* = 0.111).

**Conclusions:**

Since ulnar-sided contrast leakage is more common in patients with peripheral TFCC injuries, distinction between an atypical configuration of the prestyloid recess and actual leakage is important in CT arthrography of the wrist.

## Background

In the presence of ulnar-sided wrist symptoms, diagnostic imaging is usually focused on the triangular fibrocartilage complex (TFCC) with its articular disc, the dorsal and palmar radioulnar ligaments, as well as their peripheral insertions at the ulnar styloid process and in the ulnar fovea [1, 2]. However, despite their importance for the stability of the distal radioulnar joint (DRUJ), these components of the TFCC are not the only anatomical structures capable of causing ulnar-sided wrist pain [3]. Since the DRUJ forms a functional unit with the radiocarpal joint during pronation and supination, the entire ulnocarpal compartment participates in the stabilization of rotational movements of the hand, while simultaneously counterbalancing the transmission of axial load from the proximal carpal row to the distal forearm and vice versa [4–6]. Anatomically, the vertically oriented ulnocarpal meniscus homologue arises from the dorsoulnar edge of the articular disc and the ulnar styloid process without a visible transition zone. Its fibers converge in distal direction, attaching to the ulnopalmar surface of the triquetral bone [7]. The meniscus itself consists of dense fibrous connective tissue and its central portion contains the entrance into the ulnar prestyloid recess, which corresponds to a proximal bulge of the ulnocarpal joint space [8, 9]. In the past, the prestyloid recess has received some attention in connection to inflammatory diseases, e.g., in different forms of arthritis, which are mostly evaluated in contrast-enhanced MRI [10]. The relevance of the prestyloid recess in trauma imaging, however, has not been investigated thus far. Whereas standard computed tomography (CT) as the most common means of three-dimensional fracture analysis is unable to visualize the prestyloid recess due to a lack of articular contrast, the combination of CT imaging and direct wrist arthrography overcomes that limitation [11]. Simultaneously allowing for accurate and time efficient evaluation of bone and soft tissue, CT arthrograms have gradually replaced MRI as the standard for TFCC assessment at some institutions in recent years [12, 13]. With the excellent visibility of the prestyloid recess on CT arthrograms, the present study aims to provide a detailed analysis of its morphologic variability in a large patient group. Furthermore, since contrast leakage along the lateral side of the distal ulna is frequently seen in post-arthrographic imaging, we hypothesized that this finding is more common in association with certain injuries of the TFCC.

## Methods

### Patient population

For this retrospective study, informed consent was waived and permission obtained from the local institutional review board. Between January and December 2018, 92 consecutive patients with reported wrist trauma received CT imaging after multi-compartment arthrography of the wrist. All patients were at least 18 years old at the time of examination and assented to the procedure in written form. Six patients (three women, three men) had to be excluded from this study because the prestyloid recess could not be identified in CT scans due to a lack of contrast material. Therefore, the final study group consisted of 86 patients, including 40 women, with a median age of 44.5 years (max. 77 years; min. 18 years). The left wrist was examined in 48 patients.

### CT arthrograms

In all patients, carpal arthrography was performed by board-certified radiologists under fluoroscopic guidance using a multi-compartment approach. Before the procedure, the contrast agent (Imeron 300, Bracco Imaging) was diluted with sodium chloride for an iodine concentration of 150 mg/ml. Arthrograms were conducted as per department protocol with standardized injections into the DRUJ and the radiocarpal joint for TFCC assessment. If concomitant intrinsic ligament injuries were suspected, i.e., tears of the scapholunate and/or lunotriquetral ligament, the midcarpal joint was additionally contrasted before the injections into the more proximal articular compartments.

Immediately after the arthrography was completed, patients were transferred to the CT suite and brought in prone position with the injured wrist placed above the head in pronation for further imaging. All scans were performed with a commercially available multidetector CT system (Optima 660, GE Healthcare) using a tube voltage of 120 kVp, current–time product of 150 mAs, detector collimation of 64 × 0.6 mm and pitch factor of 1.2. Image acquisition and reconstruction was performed in axial orientation with slice thickness and increment of 0.6 mm and 0.3 mm, respectively. Using scanner-side software (Advantage Workstation, GE Healthcare), additional planes in coronal and sagittal orientation were prepared with thickness of 1 mm, increment of 0.5 mm, matrix of 1024 × 1024 pixels and field of view of 60 mm. Window width and center were preset for this study (3000 and 1000 HU), however, readers could change settings to their own demands.

### Image analysis

**Fig. 1:**
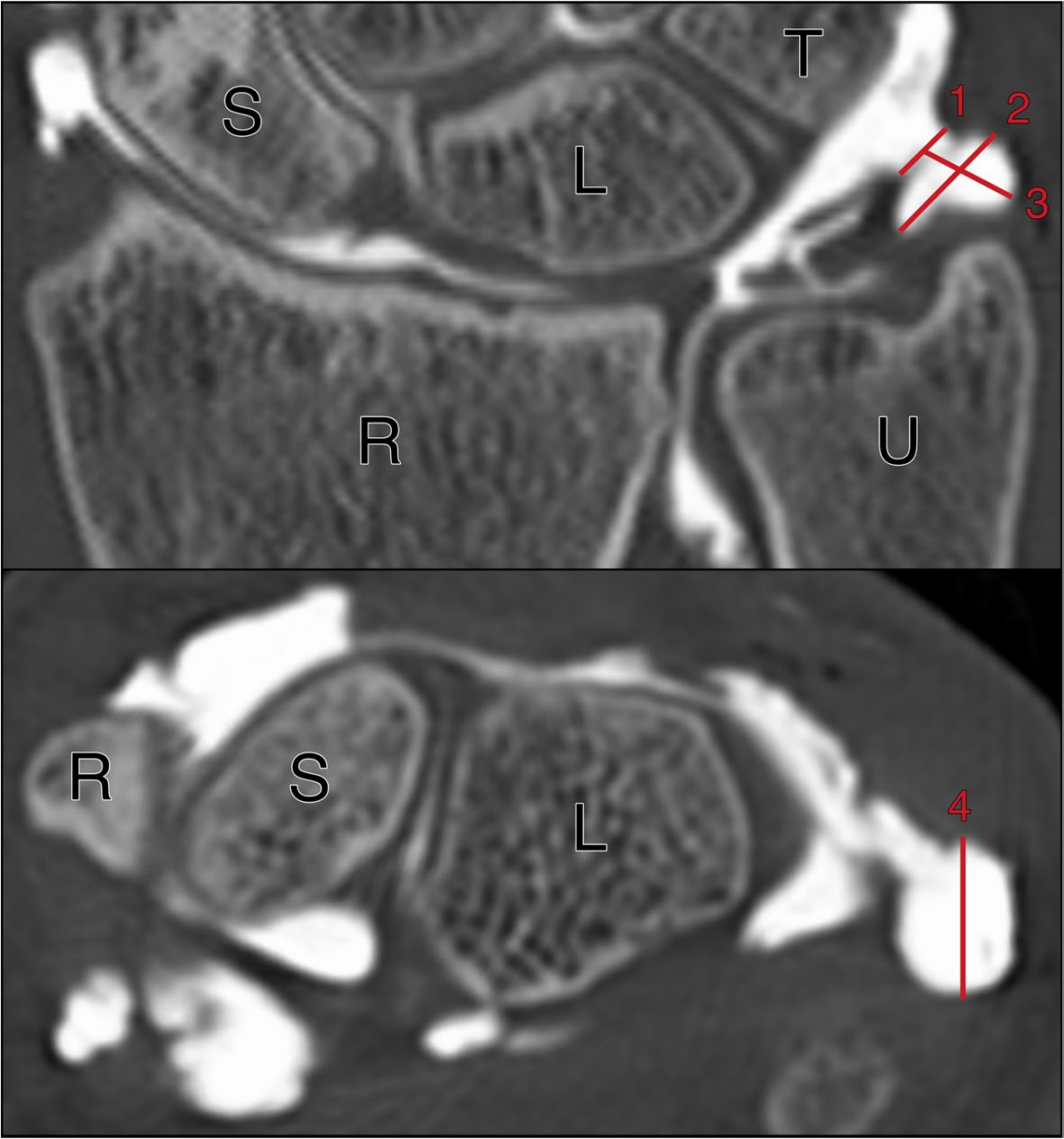
Opening width (1), bulge width (2), and maximum distance between the recess tip and opening (3) were measured in the coronal plane, while the anterior–posterior diameter of the prestyloid recess (4) was evaluated in the axial plane (S = scaphoid, L = lunate, T = triquetrum, R = radius, U = ulna)

Two board-certified radiologists with both seven years of experience in musculoskeletal imaging retrospectively analyzed all datasets in consensus on a radiologic workstation with certified diagnostic monitor (RadiForce RX660, EIZO) and dedicated PACS software (Merlin, Phönix-PACS). First, the readers were asked to qualitatively evaluate the prestyloid recess in each CT arthrogram with regard to shape (saccular, tubular, cone-shaped, tongue-shaped), opening (no opening, narrow opening, wide opening) and position (palmar, radiopalmar, ulnopalmar or apical). Second, the observers were tasked to quantify the maximum distance between the recess tip and opening, the opening width and bulge width in the coronal plane, as well as the anterior–posterior (AP) diameter of the recess in the axial plane (Fig. 1). Third, the presence of contrast leakage along the ulnar aspect of the distal ulna should be assessed in dichotomous fashion (absence or presence). Whenever any form of leakage was identified, its maximum extent in proximal direction from the distal tip of the styloid process was documented on coronal images.

For estimation of TFCC integrity, ulnar variance, and fracture involvement of the sigmoid notch and ulnar styloid process, surgical reports (available in 45 patients) were used in combination with radiological reports by musculoskeletal imaging specialists and clinical follow-up. Thereby, the latter refers to control examinations that patients were advised to undergo approximately six weeks after the initial presentation that included the CT arthrogram. For their reads, the observers were blinded to any information from clinical and radiological reports, as well as arthroscopic findings.

### Statistics

Dedicated software was utilized to conduct all statistical analyses (SPSS Statistics Version 27, IBM). Kolmogorov–Smirnov and Shapiro–Wilk tests were applied to analyze continuous variables for normal distribution. Normally distributed items are presented as means ± standard deviation, while metric data without normal distribution, as well as categorical and nominal items are reported as absolute and relative values with medians and interquartile ranges. The correlation between dichotomous variables was assessed using the mean square contingency coefficient (r_ɸ_), whereas the correlation between binary and nominal items was tested by calculation of Cramér’s V. Results were interpreted following Kotrilik et al.: 1.0–0.8: very strong; 0.8–0.6: strong; 0.6–0.4: relatively strong; 0.4–0.2: moderate; 0.2–0.1: weak; < 0.1: no association [14]. For all tests, statistical significance was presumed for p values ≤ 0.05.

## Results

Injuries of the TFCC were present in 58 patients (67.44%). Forty-six patients presented with a lesion of the articular disc (53.49%), while discontinuities of the ulnar-sided attachments were ascertained in 29 patients (33.72%). Distal radius fractures involving the sigmoid notch were recorded in 24 patients (27.91%) and 25 patients (29.07%) had suffered a fracture of the ulnar styloid process. Ulnar variance was deemed neutral in 64 cases (74.41%), whereas a substantial plus or minus variance (of at least 2 mm) was documented in 13 (22.09%) and 9 patients (10.47%), respectively. Patient characteristic are summarized in Table 1.

**Table 1:** – Patients characteristics

**Study population**	86 patients
**Sex**	
Female Male	46 (53.49%) 40 (46.51%)
**Age**	
Median Range	44.5 years 18 – 77 years
**Laterality**	
Left wrist Right wrist	48 (55.81%) 38 (44.19%)
**Ulna variance**	
Plus Neutral Minus	13 (15.12%) 64 (74.42%) 9 (10.47%)
**Fractures**	
Distal radius fractures Fractures of sigmoid notch Fractures of ulnar styloid process	47 (54.65%) 24 (27.91%) 25 (29.07%)
**Triangular fibrocartilage complex**	
Lesions overall Central lesions Peripheral lesions	58 (67.44%) 46 (53.49%) 29 (33.72%)
**Ulnar-sided contrast leakage**	
Present Absent Extent	29 (33.72%) 57 (66.28%) 12.30 ± 5.31 mm

**Fig. 2:**
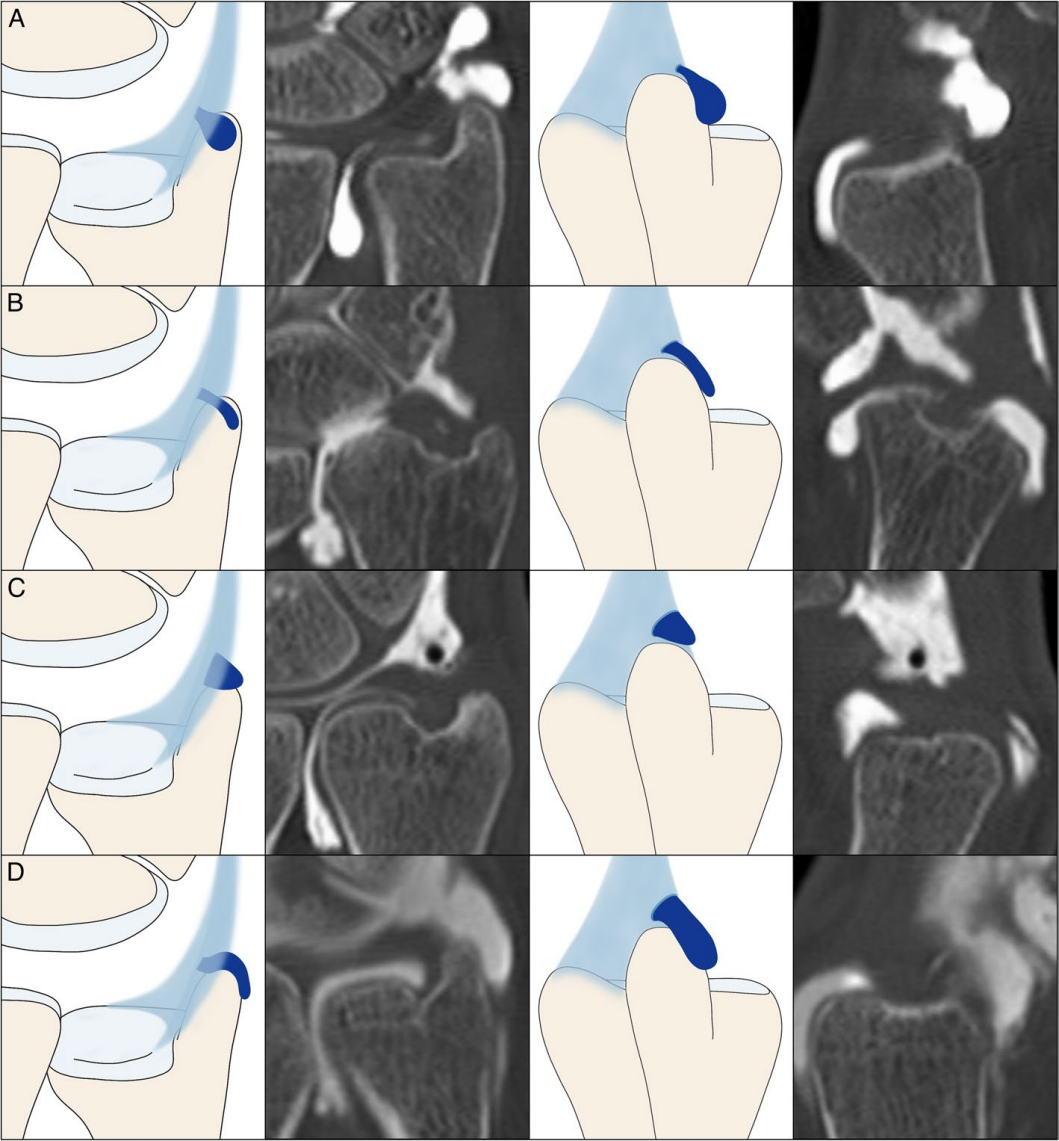
Schematic display of different prestyloid recess morphologies in coronal and sagittal planes with corresponding CT arthrograms: **A** Saccular shape. **B** Tubular shape. **C** Cone shape. **D** Tongue shape

**Fig. 3:**
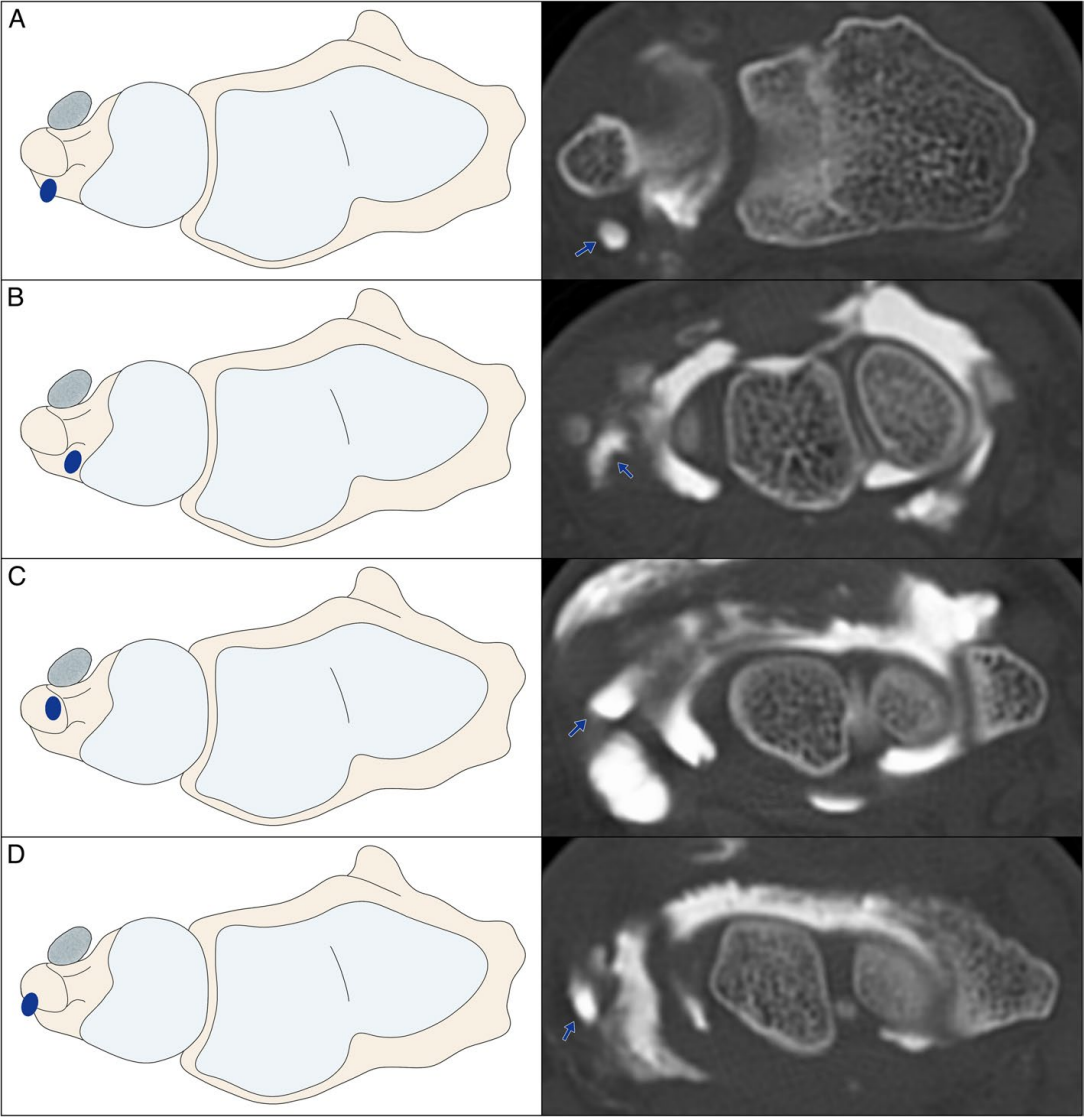
Schematic display of different prestyloid recess positions in the axial plane with corresponding CT arthrograms: **A** Palmar position. **B** Radiopalmar position. **C** Apical position. **D** Ulnoapical position

**Fig. 4:**
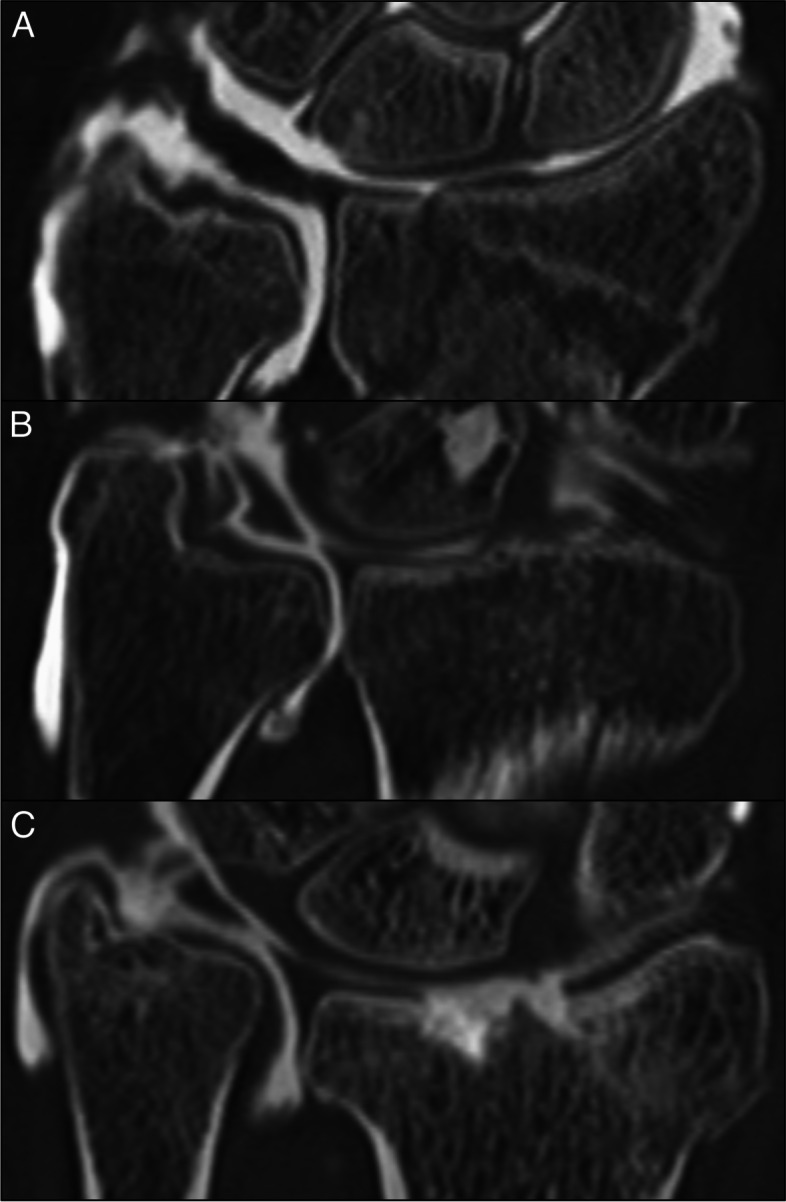
Ulnar-sided contrast leakage in different patients with traumatic lesions of the triangular fibrocartilage complex (TFCC). **A** Injury of the peripheral TFCC attachments associated with a displaced radius fracture. **B** Complex TFCC injury affecting the articular disc and the ulnar-sided periphery. **C** Impacted radius fracture resulting in ulna plus variance with an articular disc lesion and rupture of both peripheral TFCC insertions

Of 86 CT arthrograms, the opening configuration into the prestyloid recess was characterized as “narrow” in 63 (73.26%) and as “wide” in 18 patients (20.93%). Five studies did not display a distinct opening despite the presence of contrast agent in the recess (5.81%). The mean distance between recess tip and opening was 6.89 ± 2.36 mm with an opening width of 2.26 ± 1.43 mm. The recess’ shape was described as saccular, tubular, cone-shaped and tongue-shaped in 57 (66.28%), 8 (9.30%), 14 (16.28%), and 7 patients (8.14%) with a mean maximum bulge width in the coronal plane of 4.48 ± 1.94 mm (Figs. 2 and 3). In relation to the ulnar styloid process, the prestyloid recess was located palmar in 48 (55.81%), radiopalmar in 8 (9.30%), apical in 23 (26.74%) and in a ulnoapical position in 7 (8.14%) CT arthrograms (Fig. 4). The mean anterior–posterior diameter of the recess was measured with 5.05 ± 1.97 mm. A summary of ulnar prestyloid recess characteristics is included in Table 2.

**Table 2:** – Ulnar prestyloid recess morphology

**Recess opening**	
Not visible Narrow Wide	5 (5.81%) 63 (73.26%) 18 (20.93%)
**Recess shape**	
Saccular Tubular Cone Tongue	57 (66.28%) 8 (9.30%) 14 (16.28%) 7 (8.14%)
**Recess position**	
Palmar Radiopalmar Apical Ulnoapical	48 (55.81%) 8 (9.30%) 23 (26.74%) 7 (8.14%)
**Measurements**	
Opening width Bulge width Distance between recess tip and opening Anterior–posterior diameter	2.26 ± 1.43 mm 4.48 ± 1.94 mm 6.89 ± 2.36 mm 5.05 ± 1.97 mm

Ulnar-sided contrast leakage was documented in 29 patients (33.72%) with a mean extent in proximal direction from the distal tip of the ulnar styloid process of 12.30 ± 5.31 mm. Leakage was more common in patients with a documented TFCC injury than in healthy individuals (24/58 vs. 5/28; 41.38% vs. 17.86%; r_ɸ_ = 0.233; *p* = 0.031). While no association could be ascertained with the presence of central articular disc lesions (19/46 vs. 10/40; 41.30% vs. 25.00%; *p* = 0.111), ruptures of the peripheral TFCC attachments correlated in relatively strong fashion with ulnar-sided contrast leakage in CT wrist arthrography (19/29 vs. 10/57; 65.52% vs. 17.54%; r_ɸ_ = 0.480; *p* < 0.001). On the contrary, neither the presence of a styloid process fracture (*p* = 0.775), nor affliction of the sigmoid notch of the distal radius (*p* = 0.332) were associated with a higher frequency of peripheral contrast leakage. Ulnar plus (*p* = 0.096) and minus variance (*p* = 0.129), as well as recess’ opening configuration (*p* = 0.100), shape (*p* = 0.219), and position (*p* = 0.360) did also not correlate with the prevalence of ulnar-sided contrast leakage.

## Discussion

The purpose of this retrospective study was to analyze the CT appearance of the ulnar prestyloid recess after direct wrist arthrography and investigate whether ulnar-sided contrast leakage was associated with certain morphologic traits or the presence of a TFCC injury. Despite being among the most common causes of ulnar-sided wrist pain, detection and classification of TFCC lesions remains a challenge for most radiologists to this day [15]. In post-arthrographic imaging, particularly the close proximity to the contrasted ulnar prestyloid recess, which possesses high variability regarding shape and position, may lead to diagnostic uncertainty [16, 17]. The combination of multi-compartment arthrography and high-resolution CT imaging is widely established for TFCC assessment, with literature suggesting similar diagnostic accuracy as MR arthrograms [18]. While the technique of CT arthrography is less commonly in most institutions due to its invasive nature, it represents a viable alternative for patients unable to receive MRI because of contraindications. Superior contrast-to-noise ratio and articular distension are among the most striking benefits of directly injecting contrast agent into a joint compartment, aiding the differentiation between the foveal and styloid insertion of the TFCC in partial thickness tears [19]. Compared to MRI, CT arthrograms provide additional advantages, e.g., a significantly shorter examination time and a superior depiction of fracture patterns due to higher spatial resolution [20, 21].

Regarding the morphology of the ulnar prestyloid recess, we were able to show that a narrow opening (73.26%), saccular shape (66.28%) and palmar position (55.81%) are the most common characteristics. Our findings are in line with the results of H. M. Schmidt, who analyzed the ulnar-sided wrist of cadaveric specimens in an anatomical study [9]. Despite the predominance of these traits, detailed knowledge of the recess’ variable appearance is important for radiologic assessment, though. The mean extent and anterior–posterior diameter of the recess was 6.89 ± 2.36 mm and 5.05 ± 1.97 mm in our study, which is concordant with the measurements of Döring et al. and explains its excellent visibility in post-arthrographic studies [22]. However, confirming the observations of Buck et al., who studied the ulnocarpal meniscus homologue of cadaveric wrists in MRI and MR arthrography, the prestyloid recess’ size appears to be equally variable as its shape or position [8].

Ulnar leakage of contrast agent was previously described by Lee et al., who reported this phenomenon in single-compartment MR arthrography [23]. The authors found ulnar leakage in more than 50% of MR arthrograms but were unable to identify statistically significant correlations to patient data or location of symptoms. While ulnar leakage was less common in the present study, occurring in approximately one third of the patient population, we were able to show a relatively strong correlation with the presence of ulnar-sided TFCC injuries, which was not tested in the work of Lee et al. [23]. The increased frequency of ulnar leakage in lesions of the foveal and styloid attachments is in line with the findings of Machiels et al., however, who described abnormal contrast extravasation in 19 cases of dorsal peripheral detachment of the TFCC [24]. Based on our results, we believe that ulnar leakage in trauma patients may preferably occur in accompanying capsule injuries or if the TFCC is separated from its adjacent capsular elements. Since the ulnar-sided capsule is tightly adherent to the peripheral convergence of the dorsal and palmar radioulnar ligament, lesions of the ulnar-sided insertions appear to be especially predisposing for concomitant capsular injuries [25].

### Limitations

Some limitations have to be acknowledged with regard to this study. Due to the retrospective design, the patient population was heterogenous in terms of sociodemographic data and severity of trauma history. No patients without wrist pain or instability were examined, hence the prestyloid recess morphology and frequency of ulnar contrast leakage could not be compared to a healthy control group without trauma. While the inclusion of patients with distal radius fractures may have introduced a certain degree of bias, many patients that undergo CT arthrography do so specifically because of articular fractures and concomitant soft tissue injury suspicion. Considering the relatively small share of radius fractures with sigmoid notch involvement in the overall population (27.91%), we believe that the amount of patients without potential fracture-related distortions of the TFCC is sufficient. Since degenerative alterations of the central articular disc and isolated lesions of the TFCC’s styloid attachment do oftentimes not require surgery to reestablish joint stability, the number of interventions was limited in this study [26, 27]. In patients that did not undergo surgery after CT arthrography, the presence or absence of a TFCC injury was documented based on the radiological reports of musculoskeletal imaging specialists and clinical follow-up examinations if available. Since the classification of TFCC injuries according to the Palmer or Atzei system was not in the scope of our study, we decided on a simpler categorization into central and peripheral tears instead. While arthrograms were performed in pronation, ulnar variance should generally be assessed in neutral position, because the relative ulnar length is associated with the direction of forearm rotation [28]. Furthermore, rotation-dependent laxity and tightening of the palmar and dorsal radioulnar ligaments can impair the diagnostic evaluation of DRUJ stability [29].

## Conclusion

Ulnar leakage of contrast material is a common phenomenon in CT arthrograms of trauma patients, occurring most frequently in the presence of ulnar-sided TFCC injuries. While the most common configuration of the ulnar prestyloid recess includes a narrow opening, saccular shape and palmar position, precise knowledge of potential variations is important, since they can mimick aberrant contrast material in the ulnar-sided soft tissue.

## Data Availability

The datasets generated and/or analyzed during this study are not publicly available as CT data and DICOM headers contain patient information. Data can be obtained on reasonable request from the corresponding author.

## Abbreviations

DRUJ: Distal radioulnar joint
TFCC: Triangular fibrocartilage complex
